# An exploratory study of topic-specific variation in epistemic beliefs among psychology students

**DOI:** 10.3389/fpsyg.2026.1716543

**Published:** 2026-01-29

**Authors:** Lynn Adam, Machteld Vandecandelaere

**Affiliations:** Faculty of Psychology and Educational Sciences, Centre for Instructional Psychology and Technology, KU Leuven, Leuven, Belgium

**Keywords:** epistemic beliefs, epistemic thinking assessment, psychology education, TIDE framework, topic specificity, higher education

## Abstract

**Background:**

How individuals conceive knowledge and knowing plays a crucial role in psychology education. While often examined at the domain level, the Theory of Integrated Domains in Epistemology (TIDE) suggests that epistemic beliefs may also vary at the level of specific topics.

**Methods:**

We investigated whether epistemic beliefs of psychology students differ depending on the topic under consideration and tested the hypothesis that beliefs would cluster by subdisciplinary proximity (i.e., clinical vs. cognitive topics). Using the Epistemic Thinking Assessment (ETA), we implemented three scenarios addressing depression, schizophrenia, and language acquisition. A counterbalanced repeated-measures design was used with 480 first-year psychology students. Multilevel modeling was applied to distinguish topic effects from sequence effects.

**Results:**

Results indicated significant variation in epistemic beliefs across topics, leading to the rejection of the subdisciplinary hypothesis. Students scored significantly higher on absolutism and lower on evaluativism when reasoning about schizophrenia compared to depression and language acquisition. Thus, the two clinical topics did not elicit similar profiles.

**Conclusion:**

Findings confirm that epistemic beliefs are topic-specific within psychology and are driven by topic characteristics (e.g., perceived biological certainty) rather than disciplinary labels. These results highlight the need for granular, topic-specific approaches in epistemological assessments and critical thinking instruction.

## Introduction

Epistemic beliefs, that is, beliefs about the nature of knowledge and how knowledge claims are justified, are central to learning and reasoning in higher education. These beliefs shape how students evaluate theories, weigh evidence, integrate perspectives, and engage in scientific argumentation, which are core aims of psychology curricula (Hofer and Pintrich, 1997; Kuhn, 2001; Rosman et al., 2016). A student who conceives of knowledge as fixed and certain will engage differently with controversies than a student who views knowledge as contextual and justified by the best available evidence. Because such beliefs influence how learners interpret information, they also influence how instruction translates into durable understanding.

Most research distinguishes between domain-general and domain-specific conceptions of epistemic beliefs. Domain-general accounts treat epistemic beliefs as relatively stable across subjects, while domain-specific accounts assume that how students think about knowing depends on the norms and structure of a particular discipline (Schommer, 1993; Buehl and Alexander, 2001; Hofer, 2004). For example, the expectations surrounding justification and consensus in physics differ from those in psychology, and students may internalize these disciplinary differences as distinct epistemic stances. A substantial body of work supports this view by showing that epistemic beliefs covary with the perceived structure and consensus of a field (Hofer, 2004; Buehl and Alexander, 2001).

A growing line of work, however, argues for a finer level of granularity, aligned with the Theory of Integrated Domains in Epistemology (TIDE), which posits that epistemic beliefs operate at multiple reciprocal levels: general, domain-specific, and topic-specific (Muis et al., 2006; Merk et al., 2018). Rather than assuming a single stance per discipline, researchers have proposed that epistemic beliefs can vary across specific subjects and controversies within the same domain (Bråten, 2010; Kienhues et al., 2011; Urhahne and Kremer, 2023). Topic familiarity, the complexity of available explanations, and exposure to conflicting claims can all shape how learners approach knowledge claims in a given area (Kienhues et al., 2011; Merk et al., 2018). Cross-cultural research on students’ beliefs about complex issues also suggests that topic characteristics matter for how knowledge is judged (Bråten et al., 2009). If topic-level variation is robust, then assessments and interventions that treat epistemic beliefs as homogeneous within a discipline may miss important within-domain differences.

Psychology provides a particularly suitable context for studying topic-level variation. The field has been described as comparatively ill-defined, with multiple competing theories, diverse methods, and areas that differ in their degree of consensus, which makes it likely that students adjust their epistemic stance when the topic changes (Rosman et al., 2016; Klopp and Stark, 2022). Clinical topics such as depression and schizophrenia invite multi-causal explanations and ongoing debate, while topics like language acquisition, which draw on cognitive and linguistic perspectives, may be perceived as differently structured. If epistemic beliefs are sensitive to such topic characteristics, then even within psychology we should observe systematic differences in students’ stances across topics.

To examine this possibility, the present study applied the Epistemic Thinking Assessment (ETA) to three psychology topics: depression, schizophrenia, and language acquisition. Given that domain-general questionnaires often fail to capture fine-grained topic differences as predicted by the TIDE framework, a scenario-based measure is theoretically required to detect such intra-individual variation (Barzilai and Weinstock, 2015; Klopp and Stark, 2022). The ETA presents realistic, conflicting accounts of a topic and asks students to evaluate claims in terms of absolutism, multiplism, and evaluativism, which enables a context-sensitive assessment of epistemic beliefs (Barzilai and Weinstock, 2015; Klopp and Stark, 2022). We implemented a counterbalanced, repeated-measures design so that each participant completed two of the three scenarios, which allowed us to separate topic effects from potential sequence effects.

Our primary research question was whether epistemic beliefs in psychology students are topic-specific. Specifically, we tested the hypothesis that epistemic beliefs are guided by subdisciplinary proximity. We expected that beliefs would be more similar between two clinical topics (depression and schizophrenia) than between a clinical topic and a cognitive topic (schizophrenia and language acquisition). We also recorded the sequence of presentation in order to account for any order-related differences.

## Methods

### Participants

The study involved 480 first-year undergraduate psychology students from a research university in Flanders, Belgium. Participants were recruited through a university research participation pool where participation in research is a mandatory course requirement; however, students could self-select this specific study from a range of available options. The mean age of the sample was 18.73 years (SD = 1.45). Approximately 80 percent identified as female and 19 percent as male, and three participants identified as non-binary or preferred not to disclose their gender. Most participants (90.7%) had no prior higher-education degree. With regard to their secondary education track, 51.44% completed a general academic track not explicitly related to psychology, 34.37% followed a general track related to psychology, 5.54% followed a technical track related to psychology, and 8.65% reported other pathways or preferred not to answer.

### Design and procedure

We implemented a counterbalanced, repeated-measures, within-subject design. Each participant completed two of the three scenarios, with assignment to one of six counterbalanced sequences based on the final digits of the student number. This ensured that each scenario appeared equally often in the first and second position across participants. Scenarios were presented via an online survey platform. Participants completed the study in an uncontrolled setting of their own choice (e.g., at home or on campus). After reading a scenario, participants completed the corresponding ETA items. Informed consent was obtained electronically. Participation was compensated with one study credit. To avoid demand characteristics while preserving ethical standards, participants were informed about the procedures and confidentiality, while the precise theme of the study (epistemic beliefs) was disclosed after participation as part of a debriefing message.

### Instrument

We used the Epistemic Thinking Assessment (ETA), a scenario-based measure designed to elicit context-sensitive epistemic reasoning by presenting conflicting accounts and asking respondents to evaluate knowledge claims (Barzilai and Weinstock, 2015; Klopp and Stark, 2022). For psychology, we used two existing scenarios (depression and schizophrenia) previously adapted for this domain, and we developed an additional scenario on language acquisition. Each scenario was followed by 12 items mapping onto three epistemic stances: Absolutism (knowledge is certain and one correct answer exists), Multiplism (multiple answers are possible and judgments are a matter of opinion), and Evaluativism (knowledge claims should be justified by weighing evidence).

Scoring was performed by calculating the sum of responses corresponding to each epistemic stance per scenario. This resulted in three scores for each completed scenario per student (Absolutism, Multiplism, and Evaluativism), representing the frequency with which a student endorsed a particular stance. Internal consistency for the scales ranged from acceptable to good (Cronbach’s alpha ranging from 0.74 to 0.84 across scenarios). The full instrument is provided in the Appendix of the Supplementary material.

### Data analysis and power

The hierarchical structure of the data, with scenarios nested within individuals, motivated the use of multilevel modeling. We first estimated a null model to quantify the share of variance attributable to between-person differences. We then added topic as a fixed effect to test whether mean epistemic scores differed across scenarios. In a subsequent step, we added sequence position (first versus second scenario) as a fixed effect. Finally, we explored interactions between topic and sequence. Analyses were conducted in SPSS and RStudio. An *a priori* power analysis targeting a small to medium effect indicated that the achieved sample size comfortably exceeded the threshold for detecting moderate effects at conventional power.

## Results

### Descriptive findings

Table 1 presents the descriptive statistics for the three epistemic stances across topics. The general pattern was consistent across scenarios: Evaluativism yielded the highest scores, followed by absolutism, with multiplism showing the lowest endorsement. This suggests that students generally preferred evidence-based evaluations over purely opinion-based judgments. However, as shown in the multilevel analysis below, significant topic-specific shifts occurred after controlling for sequence effects.

**Table 1:** Means and standard deviations of epistemic beliefs scores by topic.

Epistemic stances	Depression	Schizophrenia	Language acquisition
Sample size (*n*)	310	333	309
Absolutism	2.82 (1.96)	3.31 (2.28)	3.41 (2.41)
Multiplism	1.20 (1.54)	1.09 (1.53)	0.93 (1.28)
Evaluativism	6.98 (2.14)	6.60 (2.28)	6.65 (2.46)

Values represent unadjusted means with standard deviations in parentheses. Scores are sum scores ranging from 0 to 12 per dimension.

### Topic-specific variation

To test the hypothesis that epistemic beliefs are topic-specific and guided by subdisciplinary proximity, we conducted multilevel models (see Supplementary Tables S1–S3 for full model parameters). These models controlled for sequence effects, which were found to be significant for absolutism.

Regarding absolutism, students scored significantly lower in the *depression* scenario than in the *schizophrenia* scenario (*b* = −0.71, *p* < 0.01). Furthermore, the multilevel model revealed that students scored higher on absolutism in the *schizophrenia* scenario than in *language acquisition* (*b* = 0.62, *p* < 0.05). Note that while raw means (Table 1) show a slightly higher score for language acquisition, the model corrects for a strong interaction between topic and presentation order, revealing the underlying trend where schizophrenia elicits higher absolutism.

Regarding evaluativism, students scored higher in the *depression* scenario than in the *schizophrenia* scenario (*b* = 0.63, p < 0.01), and higher in the *language acquisition* scenario than in *schizophrenia* (*b* = 0.86, *p* < 0.001). No significant topic effects were found for multiplism.

### Hypothesis testing

These results lead to the rejection of the hypothesis. We expected beliefs to cluster by subdisciplinary proximity (i.e., greater similarity between the two clinical topics). Instead, the data revealed that schizophrenia elicited a distinct epistemic profile—characterized by higher absolutism and lower evaluativism—compared to both depression (clinical) and language acquisition (cognitive). Thus, topic-specific features appear to outweigh general subdisciplinary categories in shaping epistemic beliefs.

## Discussion

The present study examined whether epistemic beliefs among psychology students vary with the topic under consideration. Using a counterbalanced, within-subject design, we found consistent topic effects for absolutism and evaluativism. Students reported significantly higher absolutism and lower evaluativism when reasoning about schizophrenia compared to depression and language acquisition. No reliable topic differences emerged for multiplism.

### Integrating topic-specificity into epistemic frameworks

These results confirm that epistemic beliefs are not stable, domain-general traits but are sensitive to the specific topic at hand. While domain-general and domain-specific frameworks have been indispensable for clarifying the role of epistemic cognition in learning, our findings align with the Theory of Integrated Domains in Epistemology (TIDE). This framework, recently extended by Merk et al. (2018), posits that epistemic beliefs operate at multiple reciprocal levels, including a fine-grained topic-specific level. Our data support arguments for this topic-level sensitivity and resonate with recent findings that distinct epistemic profiles exist even within related subject domains.

The fact that evaluativism was generally the highest stance across scenarios is consistent with the central role of evidence-based reasoning in psychology education. However, the topic-dependent shifts we observed indicate that this is not a fixed default. As noted by Lang et al. (2021), there is often a gap between “professed” beliefs (what students say they believe in general) and “enacted” beliefs (how they reason in a specific context). The scenario-based ETA successfully captured these enacted, context-dependent variations, which questionnaires focusing on general beliefs might miss.

### The mismatch of subdisciplinary proximity

Contrary to our hypothesis and traditional domain-specific accounts that group beliefs by discipline, we found that subdisciplinary proximity did not predict epistemic similarity. Although depression and schizophrenia are both clinical topics, they did not elicit similar epistemic profiles. Instead, schizophrenia elicited the most distinct response, characterized by higher absolutism. This finding challenges a simple mapping from disciplinary subcategory to epistemic stance and suggests that salient properties of a topic matter more than its category membership. A possible explanation is provided by Merk et al. (2018), who argue that context factors—such as topic familiarity or the nature of available explanations—can override general domain beliefs. Students may perceive schizophrenia as rooted in “hard” biological facts (e.g., genetics) compared to the multifactorial nature of depression. This aligns with research showing that students adjust their epistemic stance based on the perceived structure and consensus of the specific issue at hand.

### Educational implications

These findings have direct implications for psychology education. If students shift their epistemic stance based on the topic, instructors cannot assume that a single approach to fostering epistemological sophistication will be equally effective across all topics. First, instructors should be aware that topics perceived as fact-based may trigger a reversion to absolutist thinking. In such areas, teaching designs should explicitly surface competing theories and conflicting evidence to stimulate evaluative judgment. Second, consistent with Kuhn (2001) and Rosman et al. (2016), assignments that require structured comparison of claims and explicit justification with data can help students practice evaluative judgment. However, our results imply these interventions should be tailored to specific topics. As Urhahne and Kremer (2023) highlight, epistemic beliefs are highly specific; therefore, assessments that pool scores across dissimilar topics may conflate topic effects with individual differences.

### Limitations

Several limitations temper the conclusions. First, the study covered only three topics, two of which were clinical. Future work should broaden the topic set to include areas like social or developmental psychology to fully map the domain. Second, while we relied on an established instrument, the Language Acquisition scenario was newly developed for this study and requires further psychometric validation. Third, the study was conducted with first-year students; advanced students might show more stable profiles. Finally, data collection took place in an uncontrolled online setting. While this allowed for a large sample size, future studies might benefit from controlled lab environments to rule out distraction effects.

## Conclusion

This study provides evidence that epistemic beliefs among psychology students vary by topic within the same academic domain. Topic effects were clear for absolutism and evaluativism, and they did not align with assumptions about disciplinary subdomains. These findings support a view of epistemic beliefs as context-sensitive at the topic level and underscore the need to consider scenario content when assessing and teaching epistemic cognition. By acknowledging topic-specific variation, educators can design instruction that more effectively cultivates evaluative judgment across diverse areas.

## Data Availability

The datasets presented in this article are not readily available because the datasets generated for this study are subject to ethical restrictions. Access can be granted upon request to the authors, contingent on the approval of an addendum to the SMEC application for secondary analyses. Following such approval, anonymized data may be shared exclusively for the purpose of secondary research. Requests to access the datasets should be directed to machteld.vandecandelaere@kuleuven.be.
