# Supplementary material for: An exploratory study of topic-specific variation in epistemic beliefs among psychology students
Source: Front Psychol. 2026 Jan 29;17:1716543. doi: 10.3389/fpsyg.2026.1716543 (PMC12894252; doi:10.3389/fpsyg.2026.1716543)
Supplement: Supplementary file 1 [file Supplementary_file_1.pdf]

APPENDIX

Instrument: Epistemic Thinking Assessment

Depression Scenario

Epistemic Thinking Assessment (ETA; *Barzilai & Weinstock, 2015*)

Case adapted from Klopp & Stark (2022)

Worldwide over 350 million humans suffer from depression, which makes the disease a common and widespread illness of our time. However, the causes of depression are still unknown. The following articles seek to shed light on the causes of depression.

A scientific contribution by Prof. Dr. R. Weiland:

The main cause of depression is an excessive number of serotonin receptors in the brain. Those serotonin receptors, based in the brainstem, operate antagonistically to the receptors in the synaptic cleft. Once the serotonin molecules activate the receptors, the production of new serotonin is stopped. The more receptors the human brainstem has, the less the level to stop the serotonin production. To prove this, an experiment on mice was performed. The mice had a genetically modified number of serotonin receptors in the brainstem. Mice with an above-average number of serotonin receptors reacted barely or not at all to an SSRI, a drug that reduces the overall level of serotonin. But mice with a lower number of receptors showed a relatively fast effect. Due to this result, scientists concluded that humans with a higher number of serotonin receptors in the brainstem may be more likely to suffer from depression and react badly to antidepressants, e.g., in the form of SSRI.

A scientific contribution by Prof. Dr. W. Zimmer:

Sleep deficits can cause depression. Weariness may influence the coping of stress as well as social interaction. Furthermore, judgment and concentration are affected. Hereto, 16,000 adolescents and their parents were interviewed. Data was collected regarding symptoms of depression and suicidal ideation in association with sleep deficits. The statistical analysis concluded that the respondents who go to bed early have far less risk of suffering from depression. 24 percent of adolescents who are used to going to bed past midnight suffer more frequently from depression. Suicidal ideation was about 20 percent higher in comparison to the adolescents who are used to go to bed at about

10 pm. Regarding the sleeping schedule, 54 percent of the parents stated that their children have to be in bed by 10 pm on a school night, while 21 percent stated 11 pm and 25 percent stated (later than) midnight. Bedtime and depression correlate according to the information given by the adolescents. The risk of depression was 71 percent for all those who regularly sleep less than five hours per night in comparison to eight-hour-sleepers. Suicidal ideation occurred 48 percent more.

Please fill in the questions below about this controversy.

Questions	Response options
Is there an answer to the question of what causes depression?	a) Eventually there will be one right answer.<br>b) In principle, it is impossible to know the right answer.<br>c) There may be multiple right answers but they are not equally right.
Can there be certainty about what causes depression?	a) Eventually one could know for certain.<br>b) One could never know for certain because it is impossible to find out what happened.<br>c) There is never full certainty, but it is possible to improve the degree of certainty.
Is it possible to find out the truth about what causes depression?	a) With further investigation we would find out that there is one truth about this topic.<br>b) With further investigation we would find out that truth is in the eyes of the beholder.<br>c) With further investigation we would find out that there is more than one truth but that there are different degrees of truth.
Is there truth about what causes depression?	a) There is truth. If it is not known it is important to find it out.<br>b) There is no single truth and therefore there is no point in seeking the truth.<br>c) Truth can have many interpretations but some interpretations are better than others.
What should knowledge about depression be based on?	a) Only on the facts.<br>b) Mainly on personal points of view.<br>c) Mainly on interpretations of data.
What should knowledge about depression include?	a) Only detailed data about the topic.<br>b) Mainly people’s opinions about the topic.<br>c) Mainly theories that explain the topic.

What should be the source of knowledge of those who study depression?	a) The source of knowledge should be only in evidence that can be gathered.<br>b) The source of knowledge should be mainly in peoples' opinions and ideas.<br>c) The source of knowledge should be mainly in how people interpret the evidence that was gathered.
Does the answer to this depression controversy depend on perspectives?	a) No. One should think about this topic without being influenced by personal perspectives.<br>b) Yes. The answer to the question depends on personal perspectives.<br>c) Yes. But by considering multiple perspectives one can form a balanced position.
How should one evaluate explanations about depression?	a) The most important thing is to check if the explanations report exact data and not opinions.<br>b) The most important thing is to check if the explanations match the reader's view of the topic.<br>c) The most important thing is to check if the explanations help improve understanding of what is known about the topic.
What is the best way to judge different interpretations about this topic?	a) The best way is to check if the interpretation is based only on the facts.<br>b) The best way is to check which interpretation is most reasonable according to the reader's worldview.<br>c) The best way is to check which interpretation best explains the available data.
What would a reliable explanation be regarding this topic?	a) A reliable explanation is one that includes detailed information without opinions mixed in.<br>b) A reliable explanation is one that makes sense according to the reader's personal knowledge.<br>c) A reliable explanation is one that is based on a theory that explains the phenomena.

Schizophrenia Scenario

Epistemic Thinking Assessment (ETA; *Barzilai & Weinstock, 2015*)

Case adapted from Klopp & Stark (2022)

According to the German Federal Statistical Office, a total of 90,013 people were treated as inpatients for schizophrenia in 2014. This means that the risk of developing schizophrenia once in a lifetime is around 1%. There are various explanatory models for the causes of schizophrenia, two of which are presented below.

A specialist article by Prof. Dr. Klöbner:

Eight epidemiological twin studies have shown that the probability of both twins developing schizophrenia is significantly higher in identical twins than in fraternal twins. Epidemiological twin studies have shown that if one identical twin is diagnosed with schizophrenia, the probability that the other twin will also develop schizophrenia is approximately 40%. In fraternal twins, this probability is around 10%. Adoption studies and genetic studies on families also make it clear that the genotype underlying schizophrenia can manifest itself not only in the alternative of disease vs. no disease but can also manifest itself in so-called spectrum disorders. Spectrum disorders manifest themselves either in schizophrenia-like symptoms, e.g. schizoid personality traits, or in uncharacteristic neurotic disorders. Genetic influences can therefore be a significant cause of schizophrenia.

A specialist article by Prof. Dr. Wissmeyer:

The causes for the development of schizophrenic psychosis are often of a sociogenic nature. In the case of episodic affective disorders, it has become apparent that stressful life events often trigger the first depressive episode. A similar pattern has become apparent in schizophrenic psychoses. Although sufferers report that there is no accumulation of challenging life events or stressful situations before the first schizophrenic episode, the majority of sufferers state that they experienced a stressful life event immediately before the onset of a new schizophrenic episode.

In addition, studies have also shown that drug use, which is associated with a very high probability of stressful events or belonging to certain risk groups, can lead to the onset of schizophrenia. In this sense, drug use can therefore also represent a sociogenic factor. A further indication of

sociogenic causes is provided by the observation that neither a psychological nor a biological trigger could be detected in most relapses. It is therefore conceivable to see the causes of schizophrenia in sociogenic factors.

Please fill in the questions below about this controversy.

Questions	Response options
Is there an answer to the question of what causes schizophrenia?	a) Eventually there will be one right answer.<br>b) In principle, it is impossible to know the right answer.<br>c) There may be multiple right answers but they are not equally right.
Can there be certainty about what causes schizophrenia?	a) Eventually one could know for certain.<br>b) One could never know for certain because it is impossible to find out what happened.<br>c) There is never full certainty, but it is possible to improve the degree of certainty.
Is it possible to find out the truth about what causes schizophrenia?	a) With further investigation we would find out that there is one truth about this topic.<br>b) With further investigation we would find out that truth is in the eyes of the beholder.<br>c) With further investigation we would find out that there is more than one truth but that there are different degrees of truth.
Is there truth about what causes schizophrenia?	a) There is truth. If it is not known it is important to find it out.<br>b) There is no single truth and therefore there is no point in seeking the truth.<br>c) Truth can have many interpretations but some interpretations are better than others.
What should knowledge about schizophrenia be based on?	a) Only on the facts.<br>b) Mainly on personal points of view.<br>c) Mainly on interpretations of data.
What should knowledge about schizophrenia include?	a) Only detailed data about the topic.<br>b) Mainly people's opinions about the topic.<br>c) Mainly theories that explain the topic.
What should be the source of knowledge of those who study schizophrenia?	a) The source of knowledge should be only in evidence that can be gathered.<br>b) The source of knowledge should be mainly in peoples' opinions and ideas.

	c) The source of knowledge should be mainly in how people interpret the evidence that was gathered.
Does the answer to this schizophrenia controversy depend on perspectives?	a) No. One should think about this topic without being influenced by personal perspectives.<br>b) Yes. The answer to the question depends on personal perspectives.<br>c) Yes. But by considering multiple perspectives one can form a balanced position.
How should one evaluate explanations about schizophrenia?	a) The most important thing is to check if the explanations report exact data and not opinions.<br>b) The most important thing is to check if the explanations match the reader's view of the topic.<br>c) The most important thing is to check if the explanations help improve understanding of what is known about the topic.
What is the best way to judge different interpretations about this topic?	a) The best way is to check if the interpretation is based only on the facts.<br>b) The best way is to check which interpretation is most reasonable according to the reader's worldview.<br>c) The best way is to check which interpretation best explains the available data.
What would a reliable explanation be regarding this topic?	a) A reliable explanation is one that includes detailed information without opinions mixed in.<br>b) A reliable explanation is one that makes sense according to the reader's personal knowledge.<br>c) A reliable explanation is one that is based on a theory that explains the phenomena.

Language Acquisition

New Scenario for the Epistemic Thinking Assessment (ETA; *Barzilai & Weinstock, 2015*)

Created by Adam & Vandecandelaere, with the assistance of OpenAI (2024)

Across the globe, millions of individuals grapple with language acquisition difficulties, underscoring its prevalence as a significant challenge across diverse populations. However, the precise determinants and mechanisms driving language acquisition remain a subject of ongoing inquiry. The following articles try to elucidate the main factors influencing language acquisition.

A scientific contribution by Prof. L. Sanchez:

Language development is driven by genetic factors that influence neural pathways involved in speech and language processing. Through meticulous analysis of neuroimaging data and genetic markers, distinct patterns of brain activation and genetic predispositions associated with language acquisition were identified. Moreover, longitudinal studies have revealed that individuals with certain genetic variations exhibit accelerated language acquisition, demonstrating enhanced proficiency in linguistic tasks compared to their peers. This genetic perspective not only sheds light on the inherent differences in language development among individuals but also underscores the potential for tailored interventions designed to optimize language outcomes based on each child's unique genetic profile. By leveraging insights from genetic research, educators and clinicians can tailor interventions to address the specific needs and strengths of each child, promoting more effective language acquisition and enhancing overall communication skills.

A scientific contribution by Prof. M. Chen:

Through rigorous inquiry spanning cross-cultural comparisons and longitudinal investigations, researchers unveil the pivotal role played by environmental stimuli in shaping the linguistic development of children. It becomes evident that language acquisition is primarily influenced by various environmental factors, including the quality and quantity of parental language input, socio-economic status, and access to educational opportunities. Exhaustive statistical analyses conducted in diverse contexts reveal a compelling correlation between early exposure to rich linguistic environments and heightened language proficiency among children. This correlation underscores the crucial role of environmental stimuli in facilitating language acquisition and underscores the significance of fostering language-rich environments from an early age.

Recognizing the impact of cultural context on language acquisition underscores the importance of embracing linguistic diversity and promoting inclusive language education practices. It becomes increasingly evident that nurturing language-rich environments and providing equitable access to educational resources are essential strategies for fostering optimal language acquisition outcomes for all children, irrespective of genetic predispositions or socio-economic backgrounds.

Please fill in the questions below about this controversy.

Questions	Response options
Is there an answer to the question of how language is acquired?	a) Eventually there will be one right answer.<br>b) In principle, it is impossible to know the right answer.<br>c) There may be multiple right answers but they are not equally right.
Can there be certainty about how language is acquired?	a) Eventually one could know for certain.<br>b) One could never know for certain because it is impossible to find out what happened.<br>c) There is never full certainty, but it is possible to improve the degree of certainty.
Is it possible to find out the truth about how language is acquired?	a) With further investigation we would find out that there is one truth about this topic.<br>b) With further investigation we would find out that truth is in the eyes of the beholder.<br>c) With further investigation we would find out that there is more than one truth but that there are different degrees of truth.
Is there truth about how language is acquired?	a) There is truth. If it is not known it is important to find it out.<br>b) There is no single truth and therefore there is no point in seeking the truth.<br>c) Truth can have many interpretations but some interpretations are better than others.
What should knowledge about language acquisition be based on?	a) Only on the facts.<br>b) Mainly on personal points of view.<br>c) Mainly on interpretations of data.
What should knowledge about language acquisition include?	a) Only detailed data about the topic.<br>b) Mainly people's opinions about the topic.<br>c) Mainly theories that explain the topic.

What should be the source of knowledge of those who study language acquisition?	a) The source of knowledge should be only in evidence that can be gathered.<br>b) The source of knowledge should be mainly in peoples' opinions and ideas.<br>c) The source of knowledge should be mainly in how people interpret the evidence that was gathered.
Does the answer to this language acquisition controversy depend on perspectives?	a) No. One should think about this topic without being influenced by personal perspectives.<br>b) Yes. The answer to the question depends on personal perspectives.<br>c) Yes. But by considering multiple perspectives one can form a balanced position.
How should one evaluate explanations about language acquisition?	a) The most important thing is to check if the explanations report exact data and not opinions.<br>b) The most important thing is to check if the explanations match the reader's view of the topic.<br>c) The most important thing is to check if the explanations help improve understanding of what is known about the topic.
What is the best way to judge different interpretations about this topic?	a) The best way is to check if the interpretation is based only on the facts.<br>b) The best way is to check which interpretation is most reasonable according to the reader's worldview.<br>c) The best way is to check which interpretation best explains the available data.
What would a reliable explanation be regarding this topic?	a) A reliable explanation is one that includes detailed information without opinions mixed in.<br>b) A reliable explanation is one that makes sense according to the reader's personal knowledge.<br>c) A reliable explanation is one that is based on a theory that explains the phenomena.
