# Supplementary material for: An exploratory study of topic-specific variation in epistemic beliefs among psychology students
Source: Front Psychol. 2026 Jan 29;17:1716543. doi: 10.3389/fpsyg.2026.1716543 (PMC12894252; doi:10.3389/fpsyg.2026.1716543)
Supplement: Supplementary file 2 [file Data_Sheet_1.pdf]

Table S1

Multilevel Model for Absolutism with the three Scenarios as Reference Categories

	Schizophrenia reference category					Language reference category	Depression reference category
	Empty Model	Model 1	Model 2	Model 3	Model 4a	Model 4b	Model 4c
Fixed effects							
Intercept	3.18 (.09)***	3.31 (.12)***	2.96 (.13)***	3.24 (.16)***	3.29 (.20)***	2.67 (.22)***	2.59 (.20)***
depression scenario		-.46 (.15)**	-.41 (.14)**	-.72 (.22)**	-.71 (.24)**	-.08 (.24)	
language scenario		.07 (.15)	.06 (.14)	-.49 (.23)*	-.62 (.25)*		.08 (.24)
schizophrenia scenario						.62 (.25)*	.71 (.24)**
Second scenario			.66 (.11)***	.12 (.23)	-.03 (.24)	1.31 (.25)***	.69 (.25)**
depression*second scenario				.62 (.36)	.72 (.38).	-.63 (.38).	

lang*second scenario				1.07 (.35)**	1.35 (.38)***		.63 (.38)
schizo*second scenario						-1.35 (.38)***	-.72 (.38)
Man					-.18 (.24)	-.18 (.24)	-.18 (.24)
Previous degree non-related					.01 (.41)	.01 (.41)	.01 (.41)
Previous degree related					-.12 (.54)	-.12 (.54)	-.12 (.54)
Age					.06 (.07)	.06 (.07)	.06 (.07)
General secondary track (psychology related)					.05 (.20)	.05 (.20)	.05 (.20)
Technical secondary track (psychology related)					-.33 (.43)	-.33 (.43)	-.33 (.43)
Other secondary tracks					.08 (.36)	.08 (.36)	.08 (.36)
Random effects							
Residual	2.98 (1.73)	2.93 (1.71)	2.72 (1.65)	2.68 (1.64)	2.67 (1.63)	2.67 (1.63)	2.67 (1.63)
Intercept	2.03 (1.42)	2.02 (1.42)	2.13 (1.46)	2.14 (1.46)	2.13 (1.46)	2.13 (1.46)	2.13 (1.46)

Note. Parameter estimate standard errors listed in parentheses. * $p < .05$. ** $p < .01$. *** $p < .001$.

Table S2

Multilevel Model for Multiplism with the three Scenarios as Reference Categories

	Schizophrenia reference category					Language reference category	Depression reference category
	Empty Model	Model 1	Model 2	Model 3	Model 4a	Model 4b	Model 4c
Fixed effects							
Intercept	1.08 (.06)***	1.12 (.08)***	1.07 (.09)***	1.07 (.11)***	1.03 (.13)***	.81 (.14)***	1.11 (.13)***
depression scenario		.09 (.10)	.10 (.10)	.08 (.15)	.07 (.16)	.30 (.16)	
language scenario		-.23 (.10)*	-.23 (.10)*	-.21 (.16)	-.23 (.16)		-.30 (.16)
schizophrenia scenario						.23 (.16)	-.07 (.16)
Second scenario			.10 (.07)	.11 (.15)	.07 (.16)	.09 (.17)	.17 (.17)
depression*second scenario				.03 (.24)	.10 (.25)	.09 (.25)	
lang*second scenario				-.04 (.24)	.02 (.25)		-.09 (.25)
schizo*second scenario						-.02 (.25)	-.10 (.25)

Man					-.30 (.16)	-.30 (.16)	-.30 (.16)
Previous degree non-related					-.21 (.27)	-.21 (.27)	-.21 (.27)
Previous degree related					.07 (.35)	.07 (.35)	.07 (.35)
Age					-.01 (.04)	-.01 (.04)	-.01 (.04)
General secondary track (psychology related)					.04 (.13)	.04 (.13)	.04 (.13)
Technical secondary track (psychology related)					.53 (.28).	.53 (.28)	.53 (.28)
Other secondary tracks					.34 (.23)	.34 (.23)	.34 (.23)
Random effects							
Residual	1.30 (1.14)	1.27 (1.13)	1.27 (1.13)	1.27 (1.13)	1.17 (1.08)	1.17 (1.08)	1.17 (1.08)
Intercept	.82 (.91)	.85 (.92)	.85 (.92)	.85 (.92)	.88 (.94)	.88 (.94)	.88 (.94)

Note. Parameter estimate standard errors listed in parentheses. * $p < .05$. ** $p < .01$. *** $p < .001$.

Table S3

Multilevel Model for Evaluativism with the three Scenarios as Reference Categories

	Schizophrenia reference category					Language reference category	Depression reference category
	Empty Model	Model 1	Model 2	Model 3	Model 4a	Model 4b	Model 4c
Fixed effects							
Intercept	6.74 (.09)***	6.57 (.12)***	6.96 (.13)***	6.69 (.17)***	6.67 (.20)***	7.53 (.22)***	7.30 (.20)***
depression scenario		.36 (.15)*	.31 (.14)*	.63 (.22)**	.63 (.24)**	-.23 (.24)	
language scenario		.17 (.15)	.18 (.14)	.72 (.23)**	.86 (.25)***		.23 (.24)
schizophrenia scenario						-.86 (.25)***	-.63 (.24)**
Second scenario			-.76 (.11)***	-.22 (.23)	-.03 (.24)	-1.41 (.25)***	-.85 (.25)***
depression*second scenario				-.64 (.36).	-.82 (.38)*	.56 (.38)	
lang*second scenario				-1.04 (.36)**	-1.38 (.38)***		-.56 (.38)

schizo*second scenario						1.38 (.38)***	.82 (.38)*
Man					.48 (.25)	.48 (.25)	.48 (.25)
Previous degree non-related					.20 (.42)	.20 (.42)	.20 (.42)
Previous degree related					.05 (.56)	.05 (.56)	.05 (.56)
Age					-.05 (.07)	-.05 (.07)	-.05 (.07)
General secondary track (psychology related)					-.10 (.21)	-.10 (.21)	-.10 (.21)
Technical secondary track (psychology related)					-.20 (.44)	-.20 (.44)	-.20 (.44)
Other secondary tracks					-.42 (.37)	-.42 (.37)	-.42 (.37)
Random effects							
Residual	2.93 (1.71)	2.91 (1.71)	2.62 (1.62)	2.59 (1.61)	2.55 (1.60)	2.55 (1.60)	2.55 (1.60)
Intercept	2.34 (1.53)	2.35 (1.53)	2.49 (1.58)	2.50 (1.58)	2.39 (1.55)	2.39 (1.55)	2.39 (1.55)

Note. Parameter estimate standard errors listed in parentheses. * $p < .05$. ** $p < .01$. *** $p < .001$
